# Prevalence and Predictors of Occupational Exposure to Blood and Body Fluids Among Healthcare Workers in a Tertiary Care Hospital in Eastern India

**DOI:** 10.7759/cureus.87696

**Published:** 2025-07-10

**Authors:** Arun Mani Babu, Alok Ranjan, Chandramani Singh, Neha Chaudhary

**Affiliations:** 1 Community and Family Medicine, All India Institute of Medical Sciences, Patna, Patna, IND; 2 Community Medicine, Dr. Ram Manohar Lohia Institute of Medical Sciences, Lucknow, Lucknow, IND; 3 Community Medicine, Employees State Insurance Corporation (ESIC) Medical College and Hospital, Bihta, Patna, IND

**Keywords:** blood and body fluids, healthcare workers, hepatitis b vaccination, needle stick injury, occupational exposure, universal precautions

## Abstract

Background

Healthcare workers (HCWs) are at an increased risk of occupational exposure to blood and body fluids (BBFs), leading to transmission of blood-borne infections. Despite global data, limited information is available regarding the prevalence and predictors of such exposures in Bihar, India.

Methods

A hospital-based cross-sectional study was conducted among 250 HCWs, including resident doctors, MBBS interns, nursing staff, and paramedical staff, at All India Institute of Medical Sciences (AIIMS), Patna, from January to October 2021. Stratified random sampling was used. Data were collected using a pre-tested, semi-structured questionnaire. Descriptive statistics, chi-square test, Fisher’s exact test, and logistic regression were employed for analysis. Statistical significance was set at a p-value less than 0.05.

Results

The mean age of participants was 27 years, with 52% being female. About 22% reported at least one occupational exposure to BBFs during their duty at AIIMS Patna, and 16.4% experienced exposure in the past year. Needle stick injuries (47.3%) were the most common mode of exposure. Two-thirds of participants were fully vaccinated against hepatitis B. Following universal precautions significantly reduced the odds of exposure (adjusted odds ratio (AOR): 0.19, 95% CI: 0.09-0.40). Higher knowledge and awareness scores were also protective (AOR: 0.08, 95% CI: 0.01-0.58).

Conclusion

Occupational exposures to BBFs remain a considerable risk among HCWs. Promoting universal precaution practices, enhancing knowledge and awareness, and ensuring complete hepatitis B vaccination coverage are crucial for minimizing such risks.

## Introduction

Healthcare workers (HCWs) are an essential part of the healthcare system and play a vital role in delivering patient care. In the process, they are constantly exposed to various occupational hazards, among which exposure to blood and body fluids (BBFs) is a vital concern. These exposures place HCWs at a higher risk of acquiring blood-borne infections like hepatitis B virus (HBV), hepatitis C virus (HCV), and human immunodeficiency virus (HIV). This risk is particularly heightened in developing countries due to limitations in protective equipment, insufficient awareness, and underreporting [1,2].

Globally, the World Health Organization (WHO) estimates that more than two million such occupational exposures occur annually among HCWs, with approximately 40% of HBV and HCV infections in HCWs attributed to percutaneous injuries [1]. Even in countries like the United States, where surveillance systems exist, underreporting remains an issue, with an estimated 385,000 such cases reported each year [2-4]. A 2021 systematic review emphasized the persistent global burden and underreporting of such exposures, particularly in resource-constrained settings [2].

In India, the absence of a national reporting system and accurate surveillance mechanisms further obscures the true burden of occupational exposures. A large proportion of healthcare institutions do not maintain systematic records of such incidents. A study done in Bihar emphasized the lack of structured monitoring and highlighted that healthcare workers, especially nurses and interns, remain highly vulnerable to needlestick injuries and blood splashes [5]. With an estimated 35 lakh HCWs in India, even extrapolations from western data highlight a serious concern that remains inadequately studied [6]. Exposure can occur through a variety of routes, including percutaneous injuries like needlestick or sharps injuries, mucosal contact like splashes to the eyes or mouth, or contact with non-intact skin [3]. Poor compliance with standard precautions, like the use of personal protective equipment, hand hygiene, and safe disposal of sharps, further increases the risk. Various studies had shown that such exposures could be prevented with adequate training, improved compliance, and institutional policy enforcement [7-9].

Despite the preventable nature of many occupational exposures through proper use of universal precautions and vaccination, HCWs continue to face risks. The risk of infection following exposure depends on various factors like the type and volume of body fluid, the depth of injury, viral load in the source patient, and the immune status or vaccination status of the exposed individual. Vaccination against HBV is considered the most effective preventive measure. Yet, in many parts of India, the hepatitis B vaccination coverage remains below the recommended levels among the HCWs [7,10-12].

While several international and Indian studies have reported the prevalence and causes of occupational exposure among healthcare workers, only limited data is available from Eastern India, particularly from Bihar. This region, with its growing healthcare infrastructure and large patient load, presents unique challenges in occupational safety for HCWs. Additionally, institutional protocols for training, reporting, and managing exposure events vary widely [5,13]. So, this study was conducted in a tertiary care hospital in Eastern India to address this gap. The objectives of the study were to estimate the prevalence of occupational exposure to BBFs among HCWs and to identify the predictors associated with occupational exposure to BBFs among HCWs. The findings will be instrumental in guiding policy changes, training programs, and protective strategies to enhance occupational safety among HCWs in similar settings.

## Materials and methods

Study design, settings and study participants

This is a hospital-based cross-sectional study conducted at AIIMS, Patna, which is an Institute of National Importance located in Patna, the capital city of the state of Bihar in Eastern India. This setting was chosen as no similar study had been conducted to date in this hospital. Review of medical records had shown that accidental occupational exposure used to happen in the past. This study was carried out among the HCWs of AIIMS, Patna, from 1st January 2021 to 31st October 2021. HCWs cadres such as resident doctors, MBBS interns, nurses, and paramedical staff of AIIMS Patna, who gave consent to participate in this study, were included. 

Sample size calculation and sampling

A study conducted at an Armed Forces teaching hospital in India by Sangwan et al. reported the prevalence of needlestick injuries to be 31.43% [14]. So, the sample size calculated was 250, using Cochran’s formula where Z = 1.96 for a five percentage significance level, prevalence taken as 30%, relative error of 20%, and considering a dropout rate of 10%. Stratified random sampling using proportional allocation of study population from four different designations of HCWs such as resident doctors, MBBS interns, nursing staff, and paramedical staff, was done based on the records available on 1st January 2021, as given in Table 1.

**Table 1:** Distribution of study participants allocated to the study based on designation of healthcare workers n=250

Sl. No	Designation of healthcare workers	Total number of staff, N	Number of participants allocated for study, n
1	MBBS interns	68	11
2	Paramedical staff	216	33
3	Resident doctors	406	62
4	Nursing staff	936	144
	Total	1626	250

Study tool

The data were collected using a pre-tested, pre-designed, semi-structured questionnaire. It contains questions about their socio-demographic variables, work experience, hepatitis B vaccination status, status of universal precautions followed, history of accidental exposure, status of post-exposure prophylaxis taken, and questions to assess knowledge and awareness about occupational exposure to BBFs.

Study procedure

Randomly selected participants in each group were approached and the aim and objectives of the study were explained. Written informed consent was obtained from each participant. For the participants who consented, data were collected with a pre-tested, pre-designed, semi-structured questionnaire. The collected data were entered in Microsoft Excel student version 2019 (Redmond, WA, USA).

Study variables

The outcome of this study was to estimate the prevalence of occupational exposure to BBFs during healthcare duties at AIIMS Patna, defined as any self-reported prior incident of needlestick injury, blood splash, or other contact with potentially infectious fluids. Exposures included specific types such as needlestick injuries, blood contact through damaged skin, mucosal splash to eyes or mouth, incidents during needle recapping, and injuries during the disposal of sharps. Additional information regarding the frequency and circumstances of these exposures, such as the shift timing and number of incidents, was also collected. Key predictors examined were adherence to universal precautions such as consistent use of gloves, face shields, hand hygiene, proper sharps disposal, and avoidance of needle recapping; knowledge and awareness levels, which were assessed using a structured questionnaire and categorized into good, average, or poor based on responses; vaccination status, categorized as fully, partially, or not vaccinated for hepatitis B; professional designation, such as resident doctor, MBBS intern, nurse, or paramedical staff; and work experience, both total clinical experience and experience specific to AIIMS Patna.

Potential confounders included gender and age, as they may influence exposure risk due to variations in task assignments or risk perception; designation, which could also act as a confounder if it correlates with exposure and with adherence behavior; and shift patterns, since rotating shifts may influence fatigue and thus compliance with safety practices. The study accounted for these confounders using logistic regression analysis.

Statistical analysis

The cleaned and coded data were analyzed using SPSS version 22 (IBM Corp., Armonk, NY, USA) and Epi Info version 7 (CDC, Atlanta, GA, USA). Normality of the data was checked using a QQ plot. Data that were continuous in nature were expressed as mean and standard deviation, whereas categorical data were expressed in terms of proportion with a 95% confidence interval. The Pearson chi-square test for association was used to see the association between various independent variables and the dependent variable. Fisher's exact test was performed if the expected cell count in more than 20% of the cells was less than five. Finally, independent variables that were significant were included in the logistic regression analysis to determine the predictors of occupational exposure. The significance level was set at a p-value less than 0.05.

Ethical approval

All the study procedures were performed by the ethical standards laid down by the Institutional Ethics Committee (IEC) and by the 1964 Helsinki Declaration and its later amendments. From all those individual participants included in this study, written informed consent was obtained. Approval for this study was obtained from the Institutional Ethics Committee of AIIMS, Patna (Ref No: AIIMS/pat/IEC/PGTh/July19/28).

## Results

A total of 250 HCWs participated in this cross-sectional study, yielding a 100% response rate. The mean and median age of participants was 27 years, as given in Table 2, with a slightly higher proportion of females (52%) compared to males (48%). Designation-wise distribution showed that 57.6% were nursing staff, 24.8% were resident doctors, 13.2% were paramedical staff, and 4.4% were MBBS interns. Most of the participants had less than 24 months of experience working at AIIMS Patna (74.4%), while 55.6% had a total work experience between 24 and 60 months overall. Nearly all participants (92.8%) reported that they worked across all three duty shifts. The full hepatitis B vaccination coverage was 63.6%, whereas 17.2% were partially vaccinated, and 19.2% were not vaccinated at all. This vaccination gap represents a potential risk to HCWs in the event of accidental exposure.

**Table 2:** Distribution of study participants according to socio-demographic, work experience and hepatitis B vaccination status N=250 CI=Confidence interval AIIMS=All India Institute of Medical Sciences

SL No	Variable	Category	Frequency	Percentage (95% CI)
1	Age (in years) (Median Age = 27 years)	< 27	107	42.8 (36.8-48.4)
		≥ 27	143	57.2 (51.6-63.2)
2	Gender	Female	130	52.0 (45.6-58.3)
		Male	120	48.0 (41.7-54.4)
3	Designation	MBBS intern	11	4.4 (2.2-7.7)
		Resident doctor	62	24.8 (19.6-30.6)
		Nursing staff	144	57.6 (51.2-63.8)
		Paramedical staff	33	13.2 (9.3-18)
4	Total work experience (in months)	< 24	75	30 (24.4-36.1)
		24-60	139	55.6 (49.2-61.9)
		≥ 60	36	14.4 (10.3-19.4)
5	Work experience at AIIMS Patna (in months)	< 24	186	74.4 (68.5-79.7)
		24-60	57	22.8 (17.8-28.5)
		≥ 60	07	2.8 (1.1-5.7)
6	Work shift	Day shift	18	7.2 (4.3-11.1)
		Worked in all shifts	232	92.8 (88.9-95.7)
7	Hepatitis B vaccination status	Not Vaccinated	48	19.2 (14.5-24.6)
		Partially Vaccinated	43	17.2 (12.7-22.4)
		Fully Vaccinated	159	63.6 (57.3-69.6)

As given in Table 3, of the total participants, 55 (22%) reported at least one accidental occupational exposure to BBFs during their duty at AIIMS Patna. Among them, 41 (74.5%) reported exposure within the past one year, while the remaining exposures had occurred earlier. Among those exposed, 24 (43.6%) had only one exposure event, 22 (40%) had two exposure events, and nine (16.4%) reported three or more exposures. The most frequent type of occupational exposure was needlestick injury (47.3%), which was followed by contact with blood through damaged skin (30.9%).

**Table 3:** Distribution of study participants according to status of accidental occupational exposure to blood and body fluids ^*^N=250 ^#^N=55 ^$^The percentage is not equal to 100, as multiple responses were obtained from participants CI=Confidence interval

SL No	Status of accidental occupational exposure	Category	Frequency	Percentage (95% CI)
1	Overall accidental exposure status^*^	No	195	78.0 (72.4 - 83)
		Yes	55	22.0 (17 - 27.6)
2	Number of accidental exposures^#^	1	24	43.6 (30.3 - 57.7)
		2	22	40 (27 - 54.1)
		≥3	9	16.4 (3.5 - 45.2)
3	Accidental exposures in last one year^#^	No	14	25.5 (14.7 - 39)
		Yes	41	74.5 (61 - 85.3)
4	Source and circumstances of injury^#$^	Needle stick injury	26	47.3 (33.6 - 61.2)
		Needle recapping	13	23.6 (13.2 - 37)
		Handling of contaminated sharp devices	5	9.1 (3 - 19.9)
		Disposal in sharp containers	4	7.3 (2 - 17.6)
		Blood through damaged skin	17	30.9 (19.1 - 44.8)
		Blood through conjunctiva and mucous membranes	11	20 (10.4 - 33)
		Cleaning of instruments	7	12.7 (5.3 - 24.5)

As given in Table 4, about 75.6% of study participants were using universal precautions during their duty time. And 52.7% of study participants who had occupational exposures didn’t start the post-exposure prophylaxis within 72 hours of such exposures, and out of that, only 38.2% completed the course of post-exposure prophylaxis. About two-thirds of the study participants had obtained a good knowledge and awareness score. There was no significant association observed between the age of participants and occupational exposure categories (χ²(1) = 1.193, p = 0.275). There was no significant association observed between the gender of participants and occupational exposure categories (χ2(1) = 2.429, p = 0.119). There was a notable significant association between the designation of participants and occupational exposure categories (Fisher’s exact value = 25.89, p =< 0.001). There was a significant association between the following of universal precautions by participants and occupational exposure categories (χ2(1) = 21.61, p =< 0.001). There was also a significant association between knowledge and awareness of participants and occupational exposure categories (Fisher’s exact value = 6.045, p = 0.041). 

**Table 4:** Association of occupational exposure to blood and body fluids among the study participants with various predictor variables N=250 ^*^Statistically significant at p-value < 0.05 CI=Confidence interval

Variables	Category	Occupational exposure absent, n (%)	Occupational exposure present, n (%)	Crude odds ratio (95% CI)	p-value	Chi-square or Fisher’s exact value
Age (in years)	< 27	87 (81.3%)	20 (18.7%)	1	0.275	Chi-square: 1.193
	≥ 27	108 (75.5%)	35 (24.5%)	1.41 (0.76–2.61)		
Gender	Female	107 (82.3%)	23 (17.7%)	1	0.119	Chi-square: 2.429
	Male	88 (73.3%)	32 (26.7%)	1.7 (0.9–3.1)		
Designation	MBBS intern	7 (63.6%)	4 (36.4%)	1	<0.001*	Fisher’s exact value: 25.89
	Resident doctor	36 (58.1%)	26 (41.9%)	1.2 (0.33–4.7)		
	Nursing staff	128 (65.6%)	16 (11.1%)	0.21 (0.06–0.83)		
	Paramedical staff	24 (72.7%)	9 (27.3%)	0.65 (0.15–2)		
Universal precautions followed	No	34 (55.7%)	27 (44.3%)	1	<0.001*	Chi-square: 21.61
	Yes	161 (85.2%)	28 (14.8%)	0.21 (0.11–0.41)		
Knowledge and awareness of participants	Poor	2 (33.3%)	4 (66.7%)	1	0.041^*^	Chi-square: 6.045
	Average	62 (80.5%)	15 (19.5%)	0.12 (0.02–0.72)		
	Good	131 (78.4%)	36 (21.6%)	0.14 (0.02–0.78)		

As seen in Table 5, the logistic regression analysis of four independent variables with p-values of less than 0.2 was considered to find out the independent predictors of occupational exposure in AIIMS Patna. Only ‘following of universal precautions’ and ‘knowledge and awareness of participants’ were found to have statistical significance in preventing accidental occupational exposure after logistic regression. The statistically significant difference seen in ‘Designation’ was due to the high significance of nursing staff strata. But there was no statistical significance found for ‘designation’ after adjusting for other factors in logistic regression, as the p-value for all strata of designation was more than 0.05. Gender is not an independent predictor of accidental occupational exposure in AIIMS Patna. The Nagelkerke R² value of 21.41% indicates a moderate explanatory power of the model, and there can be other unmeasured factors that can contribute to occupational exposure risk. Since the p-value of the Hosmer and Lemeshow test is > 0.05, this model is well-fitting.

**Table 5:** Independent predictors for risk of occupational exposure among study participants N=250 ^*^Statistically significant at p-value < 0.05 Nagelkerke R²=21.41 Hosmer and Lemeshow test; Chi-square=0.287, p-value=0.866 CI=Confidence interval

Variables	Adjusted odds ratio (95% CI)	Wald test statistic	p-value
Gender			
Female	1		
Male	1.22 (0.56–2.65)	0.49	0.623
Designation			
MBBS intern	1		
Resident doctor	1.62 (0.34–7.73)	0.61	0.544
Nursing staff	0.24 (0.05–1.18)	-1.76	0.079
Paramedical staff	0.76 (0.13–4.37)	-0.31	0.756
Universal precautions followed			
No	1		
Yes	0.19 (0.09–0.40)	-4.4	<0.001^*^
Knowledge and awareness of participants			
Poor	1		
Average	0.08 (0.01–0.66)	-2.36	0.018^*^
Good	0.08 (0.01–0.58)	-2.49	0.013^*^

## Discussion

The study provides important insights about the prevalence and predictors of occupational exposure to BBFs among healthcare workers in a tertiary care hospital in Eastern India. With a 22% prevalence of occupational exposure, the findings of this study are consistent with previous studies conducted in similar tertiary care settings in India. For instance, Shriyan et al. and Kashyap et al. reported occupational exposure rates of 23.9% and 22.63%, respectively [12,15]. The slightly lower last one-year exposure rate (16.4%) reflects the increased use of personal protective equipment and training provided during the COVID-19 pandemic. Needlestick injury was the most common form of occupational exposure, aligning with global trends reported by Bouya et al. and Markovic-Denic et al. [1,13]. The predominance of these injuries highlights the need for interventions targeting sharps handling and disposal practices. Notably, over one-third of exposed workers did not initiate or complete post-exposure prophylaxis, underscoring a gap in occupational health response systems.

Hepatitis B vaccination of the HCWs is the mainstay remedy to reduce occupational HBV infection. WHO recommends that it is mandatory for all HCWs to take the hepatitis B vaccination, as they are a high-risk group [11]. Hepatitis B vaccines offer seroprotection of 85%-90% [16]. Vaccination coverage against hepatitis B was 63.6%, which, although better than earlier national averages, still leaves a significant proportion of HCWs vulnerable. Regha et al. reported higher coverage in institutions in Kerala, suggesting institutional policy differences may influence vaccination compliance [11].

The strongest predictors of occupational exposure were compliance with universal precautions and good knowledge and awareness regarding exposure risks after logistic regression analysis. These findings corroborate the conclusions of Aggarwal et al. and Kashyap et al., who emphasized education and training as critical in reducing occupational exposures [10,15]. Participants adhering to universal precautions had 81% lower odds of exposure, and those with good awareness had 92% reduced odds. Designation was not an independent predictor, although bivariate analysis indicated a higher prevalence among resident doctors and MBBS interns, possibly due to increased workload and less experience. Similar trends were observed in studies by Shokuhi et al. and Samargandy et al. [17,18]. The receiver operating characteristic (ROC) curve plotted with Nagelkerke R² = 21.41 suggests that 21.41% of the data fit this model. Since the p-value of the Hosmer and Lemeshow test is > 0.05, this model is well-fitting.

This study has several strengths. The study has a well-defined aim focusing on a relevant occupational health issue. Stratified random sampling with proportional allocation of study participants was employed to ensure representation across key HCWs cadres and achieved a 100% response rate, reducing selection bias. The use of a pre-tested, semi-structured questionnaire enhanced the reliability and validity of the data collected. Additionally, the study addresses a significant gap in the literature, especially in the context of Bihar, where data on occupational exposure among HCWs is limited.

However, the study also has limitations. Being conducted at a single tertiary care center, the findings may not be generalizable to other healthcare institutions. The inclusion was limited to four cadres of HCWs, excluding other potentially exposed groups such as housekeeping staff. Self-reported data introduces the possibility of recall bias and social desirability bias. Moreover, this study did not explore in detail the reasons behind non-compliance with universal precautions or the barriers to initiating post-exposure prophylaxis. So, future research studies should address these gaps through mixed-methods or longitudinal designs.

## Conclusions

Prevalence of occupational exposure to BBFs was found to be 22% in our study. Occupational exposure to BBFs remains a significant risk factor for HCWs in tertiary care settings, with needlestick injuries being the most prevalent mode of exposure. Adherence to universal precautions and higher knowledge and awareness levels were independently protective against such exposures. To mitigate these risks, institutional policies should prioritize comprehensive training on infection control practices, enforce consistent use of universal precautions, and ensure 100% coverage of hepatitis B vaccination among healthcare workers.

Furthermore, behavioral reinforcement, routine audits, and access to post-exposure prophylaxis are essential components of a robust occupational safety program. Future studies should include broader categories of healthcare workers and explore qualitative insights into barriers to safe practice adherence and post-exposure management.
